# Beyond patient-centered care: person-centered care for Parkinson’s disease

**DOI:** 10.1038/npjparkd.2016.19

**Published:** 2016-09-22

**Authors:** Stephen A Buetow, Pablo Martínez-Martín, Mark A Hirsch, Michael S Okun

**Affiliations:** 1 Department of General Practice and Primary Health Care, University of Auckland, Auckland, New Zealand; 2 National Center of Epidemiology and Center for Networked Biomedical Research on Neurodegenerative Diseases (CIBERNED), Madrid, Spain; 3 Department of Physical Medicine and Rehabilitation, Carolinas Medical Center, Carolinas Rehabilitation, Charlotte, NC, USA; 4 Department of Neurology, University of Florida Center for Movement Disorders and Neurorestoration, Gainesville, FL, USA

## Abstract

Interest has grown in centering Parkinson’s disease (PD) care provision on the welfare of the patient with PD. By putting the welfare of patients first, this patient-centric focus tends to subordinate the welfare of others including clinicians and carers. A possible solution is person-centered care. Rather than remove the spotlight from the patients, it widens that light to illuminate moral interests of all healthcare participants as persons whose welfare is interdependent. It assumes that unwellness among clinicians, for example, can impact the quality of the PD care they provide, such that caring for clinicians may also optimize the welfare of persons with PD. For PD, we suggest how the two models differ and why these differences are important to understand and act on to optimize benefit for participating stakeholders.

## Introduction

Care, comfort, compassion, and consolation are as important as finding a cure for Parkinson’s disease (PD). For some healthcare professionals, the source of such values is patient-centered care^1^ because ‘The good physician treats the disease; the great physician treats the patient who has the disease’.^2^ However, critics of patient-centered care view this model as less a solution than a problem because privileging the welfare of the patient can deny the same attention to the welfare of others, which may impact the quality of patient care they can provide.^3^ Under these conditions, patient-centered care can struggle to optimize care of the patient and other healthcare participants. Resolving this concern may require person-centered care*.*

Calling this latter model, ‘people-centered care,’ the World Health Organization has recognized it as a ‘major shift in thinking’ and warned against confusing it with patient-centered care.^4^ Many health systems nevertheless promote both models of care without distinguishing between them. Therefore, how the models differ and why the differences matter is poorly understood. To address these issues, we apply each model in turn to a hypothetical patient, Ann, before considering its conceptual underpinnings.

## Case study

With mid-stage PD, Ann is beginning to experience motor fluctuations and freezing episodes as well as balance dysfunction. To manage these symptoms, she takes Carbidopa–Levodopa and, to reduce its dose and prolong its effect, Entacapone. Ann’s usual PD clinician works hard to manage Ann’s PD. He has built a participatory partnership with her to enable them to co-produce patient-centered care that shares decision-making *for *Ann’s benefit. Consistent with the principles and commitments of medical professionalism,^5^ he reasons that this care satisfies the trust that society puts in him to put Ann’s welfare first. The primacy of Ann’s welfare reflects her increased health needs and diminished independence in healthcare structures in which patients have less power than clinicians. Assuming that this power asymmetry can be problematic, he respects Ann’s autonomy by maximizing her ability to make informed moral choices as the source of control^6^ in shared decisions relating to their care of her PD.

For example, Ann has asked her clinician for permission to access adjunctive hydrotherapy in an effort to improve her PD symptoms and quality of life, and decelerate her loss of functional independence. He knows there is no clear experimental evidence for the effectiveness of this intervention. Moreover, Ann’s freezing is a relative contraindication because of concern about drowning after a fall. He points to stronger evidence for functional benefits of conventional physiotherapy in PD.^7^ However, when Ann insists on trying hydrotherapy, he acknowledges that it may offer her at least short-term benefits.^8,9^ So, he agrees to her receiving it under the supervision of an experienced neurologic physiotherapist with a hydrotherapy certificate. The physiotherapist can administer the intervention in accordance with guideline recommendations^7^ during ‘on’ periods when her dopaminergic medication is optimized and works well. This compromise partly satisfies each party. It honors Ann’s preference without giving her everything she wants, given the clinician’s ambivalence toward her use of hydrotherapy. However, when he goes on vacation, Ann visits a different clinician who practises person-centered care rather than patient-centered care.

The person-centered clinician shows the same unselfish concern for Ann’s welfare. However, he understands that providing Ann with optimal patient care can require him not to neglect the moral interests of others including himself and other persons with PD. The welfare of all these persons is important to respect for the sake of their personhood and because their welfare can impact Ann. In this context, he discloses to her his lack of experience with using hydrotherapy to treat PD. He recognizes that her experience of using this intervention could therefore both benefit her and inform his capacity to share decisions with others with PD. Believing that people relate naturally to and learn from participatory story telling,^10^ he asks Ann to keep a paper or electronic diary^11^ as a ‘meaning-full’ record of what the hydrotherapy is like for her, and then share it with him.^12^ The clinician obtains relief from acting in humble good faith to explore how this care appears to impact her welfare as a whole person. He suggests that she could later choose to participate in balance and resistance training, if she wishes. It could benefit her in a manner that might also assist others within a supervised PD-specific exercise group. For the clinician, this care setting for persons with shared needs could synchronize PD care-giving and efficiently time-share the same visit with more than one patient. He adds that she might also consider taking part in a future clinical trial that may support her motor rehabilitation without compromising her right to receive the best PD care available, while potentially helping others by contributing to approved clinical research.

## Patients first

Although the meaning of patient-centered care is ‘contested and obscure’,^13^ constructions of this collaborative care approach each obligate physicians to put the welfare of patients first by directly serving these patients’ welfare interest. The approach also resonates with a commitment by physicians to the principles of patient autonomy and social justice,^5^ despite physicians lacking the means to be fully responsible for implementing them.^14^ For such reasons, patient-centered care has struggled to manage the tension between personal care and population health care and hold evidence-based medicine to critical account. Moreover, after half a century, the research evidence is weak, on balance, for clinical benefits of patient-centered care as a duty-based ethic. Although research on the safety and effectiveness of patient-centered care in PD is in its infancy, a recent Cochrane systematic review reported that patient-centered care’s ‘effects on patient satisfaction, health behavior, and health status are mixed’.^15^

An explanation for this finding is that what commonly passes as patient-centered care is unfaithful to patient-centered ideals. For example, in clinical care, patient autonomy is largely a myth. Persons with PD might slightly adjust their dosing schedules of dopaminergic drugs between visits, but they cannot determine their own medical treatments. Largely restricted to accepting or not what clinicians offer them,^16^ patients find that their moral interests are ‘honored (but not mindlessly enacted)’.^17^ Therefore, in empowering patients and their care partners (such as spouses and family members) to lead the transformation of health care through roles such as active participant peer coaches,^18^ clinicians remain in charge; indeed, even ‘expert patients’ with PD may exemplify ‘a paradox of patient empowerment and medical dominance’.^19^ Moreover, even if persons with PD could control their healthcare decisions in clinical settings, this would not fix conceptual problems with patient-centered care.

In particular, putting patient welfare first is ‘controversial at best, morally offensive at worst’.^20^ In practice, the principle invites exceptions. From surveying the literature, bioethicist David Wendler^21^ documented 27 widely acknowledged exceptions to clinicians acting in the best interests of the present patient vis-à-vis competing claims. Wendler advocated for an oversight authority to give guidance on which exceptions are legitimate and how to manage them. Yet the problem is much less a failure to provide a ‘compelling justification’ for the exceptions than how primacy of patient welfare marginalizes clinicians and others whose welfare impacts patients. Here, ‘the flaw in the metaphor (of patient-centredness) is that the patient and the doctor must coexist in a therapeutic, social, and economic relation of mutual and highly interwoven prerogatives. Neither is the king, and neither is the sun’.^22^ Our concern is that by subordinating their own welfare interest, clinicians who practise patient-centered care may contribute to widespread clinician unwellness.^23^ We are unaware of empirical research linking patient-centered care to work-related stress in clinicians. However, clinicians who devalue their self-care could compromise their personal health and thereby weaken their ability to care optimally for persons with PD.

## Persons first

Advocacy of patient-centered care for PD^1,24^ sidesteps why limitations of this care motivate the development of other care models including relationship-centered care,^25^ values-based care,^26^ whole person-care,^27^ and person-centered care. The last model recognizes at the center of health care all participants as persons whose welfare interconnects. In practice, however, there is still a tendency to interchange the terms, ‘individual,’ ‘patient’, and ‘person’^28^ and define person-centered care either as synonymous with patient-centered care or as a type of patient-centered care.^13^ The latter practice misses the point that the need to treat patients as persons is insufficient because not all persons are patients. When person-centered care speaks in an undertone to the clinician-as-person, it merely re-dresses in new clothes the model of patient-centered care that already recognizes the patient as a person. We therefore reconceptualize person-centered care to respect and balance competing moral interests and capabilities of not only the patient but also the clinician and other healthcare participants.^3^

Rather than take the spotlight off the person with PD, this model enlarges this light. In an expanded center of health care, it illuminates how all participants share the moral standing of persons. Recognizing all human beings as persons, it respects their dignity and need for caring and welfare beyond their different social roles such as patient or clinician. Persons rather than patients come first by caring for each other and themselves according to their capabilities in their particular situation. In contrast to patient-centered care as a professional duty, a virtue ethic emphasizing character development underpins such person-centered care.

The virtues are stable traits of good character that dispose persons relationally to develop their varying capabilities to make good decisions and live good lives (flourish). From our perspective, the virtues indicate not moral saintliness but rather a middle path between excess and deficiency. Thus, evident in our case study were virtues such as humility, tolerance, and good faith. It follows that person-centered PD care depends less on abstract moral principles for patient welfare than on the prior development and exercise of values and good character.

Table 1 summarizes how person-centered care differs from patient-centered care. Comparisons of attributes of these models reveal their differences. Patient-centered care emphasizes a duty to care for the health of patients first, implicitly as persons. In contrast, person-centered care makes explicit the personhood of all participatory stakeholders whose values and virtues dispose them to live the best life they can through interconnected life projects.^3^ Examples of these virtues include good faith (which expands the meaning of autonomy to include authenticity); justice as reflected in the principle of ‘equal consideration of equal interests,’ such as respect for all persons; and prudence in synthesizing science, virtue and the faith-based traditions. This integration of science with care is more balanced than patient-centered care that, until recently,^29^ has been quiet on the need for evidence to inform clinical practice. However, person-centered PD care requires comparative studies of cost and effectiveness.

In the Netherlands, a community-based professional network has reduced healthcare costs for PD^30^ but did not test our conceptualization of person-centered care. Hence, an unmet need exists to assess empirically the operational implications of resource allocation to our model. There is scope, for example, to evaluate the cost-effectiveness of ‘at home’ PD care including daily, remote monitoring of PD. Such care is growing in affordability and has the potential to improve patient access, to help clinicians recognize and treat fluctuating motor and nonmotor PD symptoms, and to ‘allow more-extensive, less-expensive participation in research’.^31^

Such evaluations should follow the implementation of our model. Its foundation in virtue ethics fits a civic theoretical perspective that requires communities to produce social arrangements for cultivating and exercising good character. Medical schools can build on the character development of recruited students through building a community of moral practice faithful to the values of person-centredness. Life-long learning can continue via self-reflection, modeling, coaching, and group discussions in organizations and systems exemplifying these moral values. These processes can be expected to help clinicians to self-care and partner with persons with PD and their caregivers so that each may benefit more equally. Together all these participants can clarify, develop, and act on deep values to protect each other and flourish. Complementing character education and social reform is the likelihood that developments in science will one day enhance personal, including moral, capabilities, and dispositions, for example, through psycho-behavioral and drug treatments.

## Figures and Tables

**Table 1:** Comparison of patient-centered care and person-centered care

*Attribute*	*Patient-centered care*	*Person-centered care*
Focus	Patient welfare	Respect for persons; reciprocated care
Personhood	Implicit	Explicit
Goal	Health maximization	Living as good a life as possible
Philosophy	Acting in socially defined, functional categories (e.g., patient) that carry rights, duties, and expectations (role theory)	Respect for the centrality and absolute value of persons as relational beings (personalism)
Ethics	Professional duty	Moral values and virtues of persons
Principles	Patients come first (primacy of patient welfare)	Persons come first
		Moral equality; equal moral interests are considered equally
	Respect for patient autonomy, especially patient agency	Authenticity, mutual agency, and bridging of competing moral interests
Care	Clinicians manage how they display their feelings (emotional labor)	Joyful and authentic care
Science	Historically quiet on science	Humanizes scientific practice
